# Needed and Received Home/Community-Based Services: Experiences of Rural Caregivers of Older Veterans

**DOI:** 10.1093/geroni/igaf122.2656

**Published:** 2025-12-31

**Authors:** Elizabeth Chamberlin, Steve Shirk, Victoria Ngo, Elizabeth Marfeo, Lauren Moo

**Affiliations:** VA Bedford Health Care System, Bedford, Massachusetts, United States; Department of Veterans Affairs, Bedford, Massachusetts, United States; VA Bedford Health Care System, Bedford, Massachusetts, United States; Tufts University, Medford, Massachusetts, United States; VA Bedford Healthcare System, Bedford, Massachusetts, United States

## Abstract

The lack of nursing homes and assisted living facilities and the wish to age at home has led to an exponential rise in informal (family, friends) caregivers. Thus, home/community-based programs and services are vital resources to keep people in their homes. As a large population of informal caregivers and care recipients live in rural locales, it is important to determine if locality matters. A national survey of informal caregivers of older (≥65) Veterans (N = 511 [rural=258, urban=253]) found no significant difference between rural and urban caregivers’ interest in types of service, with the exception that rural caregivers were more interested in respite services than urban caregivers. No difference based on location for those who actively searched for services (N = 226) (estimate = -0.11, p = 0.2058, OR = 0.80). Among the services searched for, respite was significantly different for rural and urban, with searches for this service approximately half as unlikely for those residing in urban communities, OR = 0.53. Urban caregivers reported receiving caregiver support, community, and helping services more than rural caregivers, with urban caregivers approximately three times as likely to have received support (OR = 3.01) and over twice as likely to have received community and helping services (OR = 2.35 and OR = 2.32, respectively) than their rural counterparts. These findings highlight the unevenness in services needed and those received by caregivers’ locality. The lack of services in rural areas can decrease the overall health of older rural Veterans and lead to institutionalization.

